# Neurological improvement is associated with neck pain attenuation after surgery for cervical ossification of the posterior longitudinal ligament

**DOI:** 10.1038/s41598-021-91268-2

**Published:** 2021-06-07

**Authors:** Masao Koda, Toshitaka Yoshii, Satoru Egawa, Kenichiro Sakai, Kazuo Kusano, Yukihiro Nakagawa, Takashi Hirai, Kanichiro Wada, Keiichi Katsumi, Atsushi Kimura, Takeo Furuya, Satoshi Maki, Narihito Nagoshi, Kota Watanabe, Tsukasa Kanchiku, Yukitaka Nagamoto, Yasushi Oshima, Kei Ando, Hiroaki Nakashima, Masahiko Takahata, Kanji Mori, Hideaki Nakajima, Kazuma Murata, Shunji Matsunaga, Takashi Kaito, Kei Yamada, Sho Kobayashi, Satoshi Kato, Tetsuro Ohba, Satoshi Inami, Shunsuke Fujibayashi, Hiroyuki Katoh, Haruo Kanno, Hiroshi Takahashi, Kengo Fujii, Masayuki Miyagi, Gen Inoue, Masashi Takaso, Shiro Imagama, Yoshiharu Kawaguchi, Katsushi Takeshita, Masaya Nakamura, Morio Matsumoto, Atsushi Okawa, Masashi Yamazaki

**Affiliations:** 1 Department of Orthopedic Surgery, Faculty of Medicine, University of Tsukuba, 1-1-1 Tennodai, Tsukuba, Ibaraki 305-8575 Japan; 2 Department of Orthopedic Surgery, Tokyo Medical and Dental University, 1-5-45 Yushima, Bunkyo Ward, Tokyo, 113-8519 Japan; 3 Department of Orthopedic Surgery, Saiseikai Kawaguchi General Hospital, 5-11-5 Nishikawaguchi, Kawaguchishi, Saitama 332-8558 Japan; 4 Department of Orthopedic Surgery, Kudanzaka Hospital, 1-6-12 Kudanminami, Chiyadaku, 102-0074 Japan; 5 Department of Orthopaedic Surgery, Wakayama Medical University Kihoku Hospital, 219 Myoji, Katsuragi-cho, Itogun, Wakayama 649-7113 Japan; 6 Department of Orthopedic Surgery, Hirosaki University Graduate School of Medicine, 5 Zaifucho, Hirosaki, Aomori 036-8562 Japan; 7 Department of Orthopedic Surgery, Niigata University Medicine and Dental General Hospital, 1-754 Asahimachidori, Chuo Ward, Niigata, Niigata 951-8520 Japan; 8 Department of Orthopedics, Jichi Medical University, 3311-1 Yakushiji, Shimotsuke, Tochigi 329-0498 Japan; 9 Department of Orthopedic Surgery, Chiba University Graduate School of Medicine, 1-8-1 Inohana, Chuo Ward, Chiba, Chiba 260-0856 Japan; 10 Department of Orthopaedic Surgery, School of Medicine, Keio University, 35 Shinanomachi, Shinjuku Ward, Tokyo, 160-8582 Japan; 11 Department of Orthopedic Surgery, Yamaguchi University School of Medicine, 1144 Kogushi, Ube, Yamaguchi 755-8505 Japan; 12 Department of Orthopedic Surgery, Osaka Rosai Hospital, 1179-3 Nagasonecho, Sakaishi, Osaka 591-8025 Japan; 13 Department of Orthopaedic Surgery, Faculty of Medicine, The University of Tokyo, 7-3-1 Hongo, Bunkyo-ku, Tokyo, 113-0033 Japan; 14 Department of Orthopedic Surgery, Nagoya University Graduate School of Medicine, 65 Tsurumaicho, Showa Ward, Nagoya, Aichi 466-8550 Japan; 15 Department of Orthopaedic Surgery, Faculty of Medicine and Graduate School of Medicine, Hokkaido University, Kita 15, Nishi 7, Sapporo, 060-8638 Japan; 16 Department of Orthopaedic Surgery, Shiga University of Medical Science, Tsukinowa-cho, Seta, Otsu, Shiga 520-2192 Japan; 17 Department of Orthopaedics and Rehabilitation Medicine, Faculty of Medical Sciences, University of Fukui, 23-3 Matsuoka Shimoaizuki, Eiheiji-cho, Yoshida-gun, Fukui 910-1193 Japan; 18 Department of Orthopedic Surgery, Tokyo Medical University, 6-7-1 Nishishinjuku, Shinjuku-ku, Tokyo, 160-0023 Japan; 19 Department of Orthopedic Surgery, Imakiire General Hospital, 4-16 Shimotatsuocho, Kagoshimashi, 892-8502 Japan; 20 Department of Orthopedic Surgery, Graduate School of Medicine, Osaka University, 2-2 Yamadaoka, Suita-shi, Osaka, 565-0871 Japan; 21 Department of Orthopaedic Surgery, Kurume University School of Medicine, 67 Asahi-machi, Kurume-shi, Fukuoka, 830-0011 Japan; 22 Department of Orthopedic Surgery, Hamamatsu University School of Medicine, 1-20-1 Handayama, Hamamatsu, Shizuoka 431-3125 Japan; 23 Department of Orthopaedic Surgery, Graduate School of Medical Sciences, Kanazawa University, 13-1 Takara-machi, Kanazawa, 920-8641 Japan; 24 Department of Orthopedic Surgery, University of Yamanashi, 1110 Shimokato, Chuo Ward, Yamanashi, 409-3898 Japan; 25 Department of Orthopaedic Surgery, Dokkyo Medical University School of Medicine, 880 Kitakobayashi, Mibu-machi, Shimotsuga-gun, Tochigi, 321-0293 Japan; 26 Department of Orthopaedic Surgery, Graduate School of Medicine, Kyoto University, 54 Kawahara-cho, Shogoin, Sakyo-ku, Kyoto, 606-8507 Japan; 27 Department of Orthopedic Surgery, Surgical Science, Tokai University School of Medicine, 143 Shimokasuya, Isehara, Kanagawa 259-1193 Japan; 28 Department of Orthopaedic Surgery, Tohoku University School of Medicine, 1-1 Seiryomachi, Aoba Ward, Sendai, Miyagi 980-8574 Japan; 29 Department of Orthopedic Surgery, Faculty of Medicine, University of Toyama, 2630 Sugitani, Toyama, Toyama 930-0194 Japan; 30 Department of Orthopaedic Surgery, Kitasato University School of Medicine, Minami Ward, 1-15-1 Kitasato, Sagamihara, Kanagawa 252-0375 Japan; 31 Japanese Multicenter Research Organization for Ossification of the Spinal Ligament, Tokyo, Japan

**Keywords:** Chronic pain, Spinal cord diseases, Outcomes research

## Abstract

Although favourable surgical outcomes for myelopathy caused by cervical ossification of the posterior longitudinal ligament (OPLL) have been reported, factors significantly associated with post-operative neck pain attenuation still remain unclear. The primary aim of the present study was to determine factors significantly associated with post-operative neck pain attenuation in patients with cervical OPLL using a prospective multi-centre registry of surgically treated cervical OPLL. Significant postoperative neck pain reduction (50% reduction of neck pain) was achieved in 31.3% of patients. There was no significant difference in neck pain attenuation between surgical procedures. Statistical analyses with univariate analyses followed by stepwise logistic regression revealed neurological recovery as a factor having a significant positive association with post-operative neck pain attenuation (*p* = 0.04, odds ratio 5.68 (95% confidence interval: 1.27–22.2)). In conclusion, neurological recovery was an independent factor having a significant positive association with post-operative neck pain attenuation in patients with cervical myelopathy caused by OPLL who underwent cervical spine surgery.

## Introduction

Ossification of the posterior longitudinal ligament (OPLL) is a disease with heterotopic ossification in the spinal posterior longitudinal ligament^1^. Computed tomography screening has revealed an unexpectedly high prevalence of OPLL in the cervical spine, which affects about 7–10% of the general population in Japan^2,3^. Increase of thickness of ossification foci can cause compression of nerve roots and the spinal cord, possibly resulting in neurological deficits^4^. Favourable surgical outcomes for cervical OPLL have been reported. Nerve root and spinal cord symptoms including numbness, palsy, and vesico-rectal disturbance can be attenuated after surgery for cervical OPLL^5^.

In addition to neurological symptoms including radiculopathy and myelopathy, cervical OPLL can cause local symptoms such as neck pain and stiffness, which can be main complaints for patients^6^. Previous reports revealed neck pain attenuation by cervical spine decompression/fusion surgeries for cervical OPLL^7–10^. However, the precise aetiology of neck pain, postoperative change of neck pain, and factors significantly associated with post-operative neck pain attenuation in patients with cervical OPLL still remain unclear.

The primary aim of the present study was to elucidate the post-operative change of neck pain and to determine factors significantly associated with post-operative neck pain attenuation in patients with cervical OPLL using a prospective multi-centre registry of surgically treated cervical OPLL.

## Results

Patient demographics are shown in Table 1. Pre-operative visual analogue scale (VAS 1–100 mm) neck pain score was 62.0 ± 21.4 mm on average (± SD), VAS neck pain score was reduced to 46.2 ± 27.2 mm at 1 year after surgery, therefore, post-operative 1 year VAS neck pain score reduction after surgery was 15.9 ± 26.1 mm and VAS neck pain score reduction was 23.1 ± 46.6%, and VAS neck pain score 2 years after surgery was 48.2 ± 28.8 mm, post-operative 2 years VAS neck pain score reduction was 13.8 ± 28.4 mm and VAS neck pain score reduction was 18.6 ± 53.4%. Fifty-six patients (21.1%) showed post-operative neck pain deterioration, whereas the remaining 209 (78.9%) showed no deterioration. Significant attenuation of neck pain, which was set to 50% reduction of VAS neck pain, was achieved in 77 out of 265 patients 1 year after surgery (29.1%) and 83 out of 265 patients 2 year after surgery (31.3%, Table 2).

**Table 1:** Patient demographics.

Demographics (n = 265)	
Male: Female (cases)	194: 71
Age at surgery (years old)	63.3 ± 11.7
Disease duration (months)	43.1 ± 59.7
Body mass index	25.7 ± 4.2
Diabetes (no. of cases)	87/265
JOA score (pts.)	
Pre-op	10.4 ± 3.1
Post-op. 1y	13.6 ± 2.6
Post-op. 2y	13.4 ± 2.9
Acquired points	3.0 ± 2.7
Recovery rate (%)	44.9 ± 35.5
Pre-op. neck pain (VAS, mm)	62.0 ± 21.4
Surgical procedures (cases)	
Laminoplasty	145
Posterior decompression & fusion	64
Anterior decompression & fusion	56
Imaging findings	
Type of OPLL (cases)	
Continuous	30
Segmental	99
Mixed	115
Localized	21
Canal narrowing rate (%)	43.5 ± 15.6
C2-7 angle (°)	9.3 ± 11.9 (ΔC2-7 angle: − 1.7 ± 9.9)
range of motion (°)	26.9 ± 14.0 (ΔROM: − 9.6 ± 15.1)
T2 high signal change (cases)	231/265

**Table 2:** Postoperative change of neck pain in overall participants.

Neck pain (VAS, 0-100 mm)	
Pre-op	62.0 ± 21.4 mm
Post-op. 1y	46.2 ± 27.2 mm
VAS reduction	15.9 ± 26.1 mm
VAS reduction rate	23.1 ± 46.6%
50% pain reduction (cases)	77/265 (29.1%)
Post-op. 2y	48.2 ± 28.8 mm
VAS reduction	13.8 ± 28.4 mm
VAS reduction rate	18.6 ± 53.4%
50% pain reduction (cases)	83/265 (31.3%)

Pre-operative visual analogue scale (VAS 1–100 mm) neck pain score was 62.0 ± 21.4 mm on average (± SD), VAS neck pain score was reduced to 46.2 ± 27.2 mm at 1 year after surgery, therefore, post-operative 1 year VAS neck pain score reduction after surgery was 15.9 ± 26.1 mm and VAS neck pain score reduction was 23.1 ± 46.6%. VAS neck pain score 2 years after surgery was 48.2 ± 28.8 mm, post-operative 2 years VAS neck pain score reduction was 13.8 ± 28.4 mm and VAS neck pain score reduction was 18.6 ± 53.4%. Significant attenuation of neck pain, which was set to 50% reduction of VAS neck pain, was achieved in 77 out of 265 patients 1 year after surgery (29.1%) and 83 out of 265 patients 2 year after surgery (31.3%).

There was no significant difference in pre-operative VAS neck pain score between surgical procedures. Post-operative VAS neck pain scores, neck pain reduction score, and proportion of neck pain reduction showed no significant difference between surgical procedures (Table 3).

**Table 3:** Comparison of postoperative neck pain reduction and JOA score between surgical procedures.

	LMP	PDF	ADF
	(n = 145)	(n = 64)	(n = 56)
Neck pain			
Pre-Op. (mm)	60.0 ± 22.4	64.0 ± 20.5	65.1 ± 20.0
Post-Op. (mm)	48.0 ± 28.3	47.6 ± 30.0	49.4 ± 29.7
Change (mm)	11.9 ± 27.5	16.3 ± 30.1	15.9 ± 28.8
Reduction rate (%)	16.0 ± 55.4	21.9 ± 51.3	21.7 ± 51.0
50% pain red. (cases)	41/145 (28.3%)	22/64 (34.4%)	20/56 (35.7%)
JOA score			
Pre-Op. (pts.)	11.0 ± 2.7	8.8 ± 3.5	11.0 ± 2.7
Post-Op. (pts.)	13.7 ± 2.5	12.2 ± 3.6	14.1 ± 2.6
Change (pts.)	2.6 ± 2.4	3.4 ± 3.3	3.3 ± 2.6
Recovery rate (%)	42.6 ± 36.6	42.3 ± 34.3	54.3 ± 33.0

There was no significant difference in neck pain attenuation and JOA score recovery between laminoplasty, PDF and ADF.

Age, diabetes mellitus, disease duration and Japanese Orthopedic Association score for evaluating cervical myelopathy (JOA score) recovery rate were identified as possible candidates for factors having significant association with postoperative neck pain attenuation by initial uni-variate analyses (Table 4). Logistic regression analysis revealed the JOA score recovery rate as an independent factor having a significant positive association with post-operative neck pain attenuation (*p* = 0.04, odds ratio 5.68 (95% confidence interval: 1.27–22.2), Table 4). Receiver-operator characteristic (ROC) analysis revealed a JOA score recovery rate of 52.6% as a cut-off value to achieve at least a 50% reduction of post-operative neck pain score (area under curve (AUC) = 0.6).

**Table 4:** Statistical analyses.

Univariate analysis	*p*-value (#: p < 0.1)	
Patients factors		
Age	0.04^#^	
Sex	0.36	
Body mass index	0.20	
Disease duration	0.07^#^	
Diabetes mellitus	0.07^#^	
Neurological status		
Pre-op. JOA score	0.56	
Post-op. JOA score	0.15	
JOA score change	0.11	
JOA score recovery rate	0.02^#^	
Neck pain		
Pre-op. neck pain	0.52	
Imaging factors		
OPLL types	0.19	
Canal narrowing rate	0.67	
C2-7 angle change	0.68	
C2-7 range of motion change	0.38	
MRI T2WI signal change	0.30	

Stepwise Logistic Regression	*p*-value (*: *p* < 0.05)	Odds ratio (95% confidence interval)
Age	0.05	
Disease duration	0.08	
Diabetes mellitus	0.07	
JOA score recovery rate	0.04*	5.68 (1.27–22.2)

Age, diabetes mellitus, disease duration and JOA score recovery rate were identified as possible candidates for factors having significant association with postoperative neck pain attenuation by initial uni-variate analyses. Stepwise logistic regression analysis revealed the JOA score recovery rate as an independent factor having a significant positive association with post-operative neck pain attenuation (*p* = 0.04, odds ratio 5.68 (95% confidence interval: 1.27–22.2), Table 4).

## Discussion

The present results demonstrated that neurological recovery was an independent factor having a significant positive association with post-operative neck pain attenuation.

There are many previous reports showing the possible aetiologies of neck pain.

Axial pain, as first reported by Hosono, which is defined as post-operative neck pain related to posterior approach-induced muscle damage, is regarded as a major cause of post-operative neck pain^11^. Various kinds of muscle preserving posterior approaches have been reported to attenuate post-operative axial neck pain^12–14^. The anterior approach does not invade the posterior musculo-ligamentous complex; therefore, post-operative muscle-related neck pain can be decreased compared with that in the posterior approach^15^. However, the present results showed that there is no significant difference in post-operative neck pain attenuation between surgical approaches (anterior and posterior) or surgical procedures (laminoplasty, PDF and ADF), suggesting that surgical damage of the cervical musculature has no significant association with post-operative neck pain in the present patient series. Possible explanations for this discrepancy in muscle damage-related neck pain between previous reports and the present data might be as follows: the posterior approach-related muscle damage decreased according to the recent popularization of muscle-preserving posterior approaches and the impact of posterior approach-related muscle damage might be limited to the early post-operative phase and not the chronic phase.

Discogenic and/or facet genic neck pain, which is caused by degenerated intervertebral disks and facet joints accompanied with segmental instability, can be another possible source of neck pain^16–18^. Fusion surgery can be indicated for discogenic/facet genic neck pain because this category of pain can theoretically be attenuated by fusion of the pain-generating segment^19^. However, the present results unexpectedly showed that there was no significant difference in post-operative neck pain attenuation between segmental motion-preserving laminoplasty and fusion surgeries (anterior and posterior). Therefore, discogenic/facet genic neck pain was not likely to be a major aetiology of neck pain in the present series.

The present results revealed post-operative neurological recovery as an independent factor having a significant association with post-operative neck pain attenuation. These lines of evidence suggest that neurogenic pain is one of the major causes of neck pain in patients with cervical OPLL. There might be several possible origins of myelopathy-related neck pain. Spinal cord compression can stimulate the posterior ramus of the spinal nerve, possibly resulting in neck pain^19^. Segmental spinal cord sign caused by compressive myelopathy may, like girdle pain, be another origin of neck pain^20^. Segmental spinal cord compression can cause local impairment of the spinothalamic tract at its chiasma at the central grey matter of the spinal cord^21^. Irrespective of the precise cause, a large-scale cohort study revealed that cervical myelopathy can cause neck pain^22^.

In addition, there is a possibility that the natural course of the disease is attributed to postoperative neck pain attenuation. Theoretically, progression of ossification foci can lead to spontaneous fusion of intervertebral segments, potentially resulting in neck pain attenuation. However, we could not find articles describing the natural history of neck pain in OPLL patients. Therefore, we had no proper answer for this question at present. Our multicenter study group is now constructing another prospective registry for OPLL patients receiving non-operative treatment with long term follow-up. Precise natural course of OPLL might be elucidated in future.

The present study includes several major limitations. The present registry lacks data regarding cervical sagittal alignment. Recently, the concept of sagittal alignment has been introduced to the cervical spine, similar to the thoracolumbar spine. Cervical sagittal alignment is important to evaluate neck pain because it has been reported to correlate with neck pain^23^. Therefore, the outcome might be changed significantly if cervical sagittal alignment data were added. To solve this problem, future collection of data regarding cervical sagittal alignment is needed. Another major limitation of the present study is that the present registry lacks information about the precise location and characteristics of neck pain and evaluation of neuropathic pain. Those data are important to elucidate the origin of neck pain. As a result, we can only speculate on the origin of neck pain using indirect evidence including post-operative change of neck pain, pre-operative patient factors, surgical factors, radiological changes, and neurological status. Future data collection of the precise characteristics of neck pain and neuropathic pain evaluation are warranted.

In conclusion, neurological recovery was an independent factor having a significant positive association with post-operative neck pain attenuation in a prospective study of a cohort of patients with cervical myelopathy caused by OPLL who underwent cervical spine surgery.

## Methods

We used a prospective cohort design for the present study.

We assembled as investigator’s meeting before the initiation of the present study and twice a year during the study period for training to standardize the data collection and imaging analyses. All the clinical and imaging data were collected by physicians except for surgeons who performed surgery in each institute. Cleaning of collected data was performed by the committee member of the present study group. Missing data was mainly caused by patients’ drop out from follow-up.

The registry included data from 478 patients who underwent cervical spine surgery for myelopathy caused by cervical OPLL. Amongst these patients, we excluded data from those who lacked pre-operative neck pain evaluation (40 cases), who showed a pre-operative neck pain score < 30 mm on a visual analogue scale (VAS, 0–100 mm, 166 cases) to avoid possible ceiling effects in evaluation for pain attenuation and several previous pain trials set similar exclusion criteria^24^. We also excluded patients received anterior–posterior combined surgery because the number of those patients was very small (n = 7) to obtain statistical significance.

Therefore, we included data from 265 patients with cervical OPLL and a pre-operative neck pain severity score ≥ 30 mm on a VAS. Patient demographics are shown in Table 1.

Neck pain was evaluated using the VAS score pre- and post-operatively. The proportion of VAS score reduction was calculated as (pre-operative VAS neck pain—post-operative VAS neck pain) / pre-operative VAS neck pain × 100 (%). Post-operative neck pain deterioration was expressed as the negative proportion of VAS score reduction. We employed “50% pain reduction” as classification for sufficient postoperative neck pain attenuation because we think it is comprehensive at a glance and it is one of the popular outcomes in pain research field^24^. In addition, another reason why we adopted 50% pain reduction as the outcome measure is that the impact of absolute value of pain evaluation could differ between patients having different preoperative neck pain.

Possible explanatory factors having significant association with postoperative neck pain attenuation were as followings.

*Patient factors* Patients factors included age at surgery, sex, body mass index, disease duration, and diabetes mellitus.

*Neurological status* Pre- and post-operative neurological status were analysed using the Japanese Orthopedic Association score for evaluating cervical myelopathy (JOA score; 0–17 points^25^). The recovery rate of JOA score was calculated using the following method: (post-operative JOA score—pre-operative JOA score) / (17 (full mark)—pre-operative JOA score) × 100 (%)^26^. Post-operative neurological deterioration was expressed as the negative value JOA score recovery rate.

**Figure 1:**
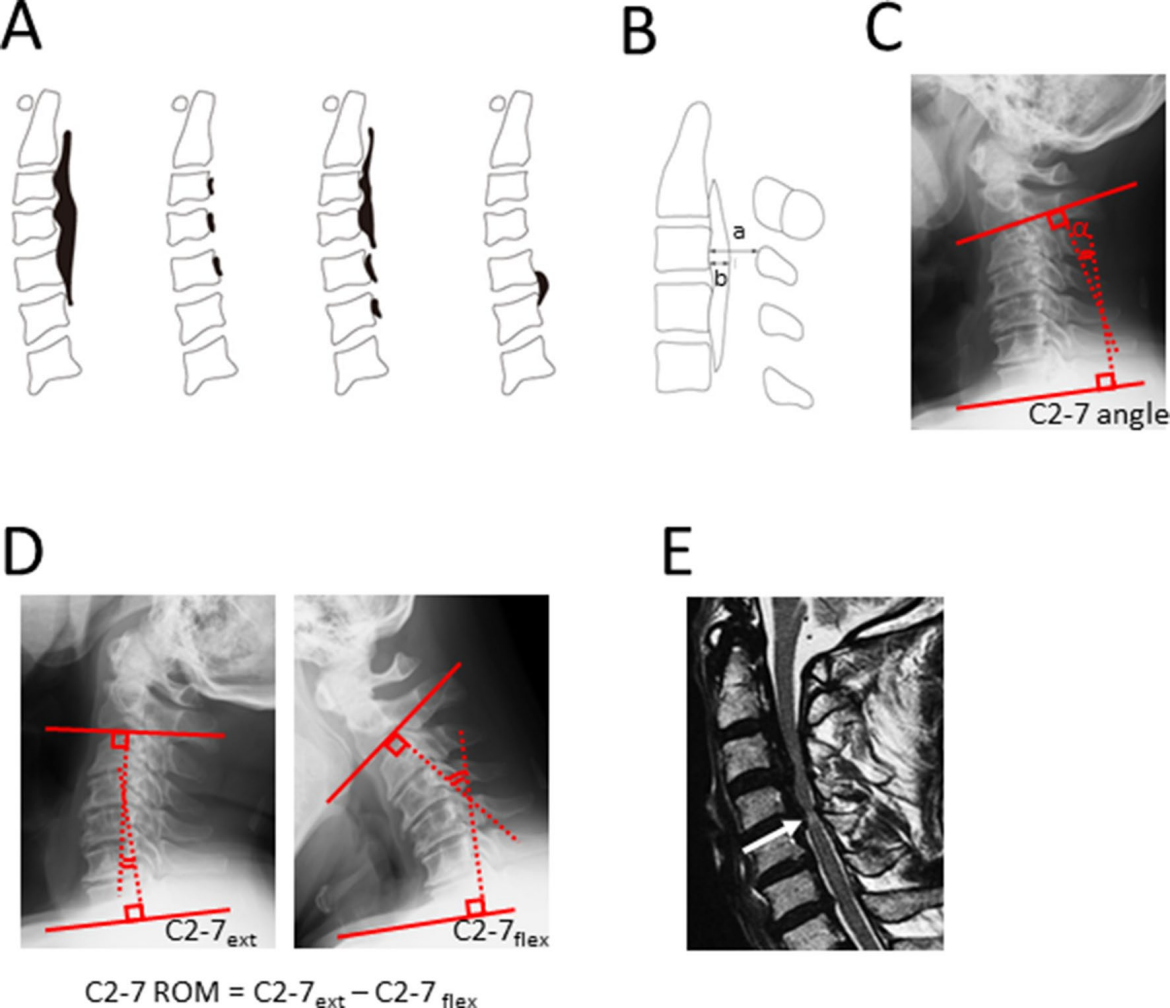
Methods for imaging analyses. (**A**) OPLL was classified into 4 types (continuous, segmental, mixed and localized types). (**B**) Canal narrowing rate was calculated as followings, thickness of OPLL at peak (b)/antero-posterior diameter (a), in lateral radiogram of cervical spine in neutral position. (**C**) C2-7 angle was measured as angle between inferior endplates of C2 and C7 vertebral bodies (**C**). Lordosis curve was expressed as positive value and kyphosis curve was expressed as negative value. Post-operative change of C2-7 angle was calculated as subtraction of postoperative C2-7 angle from preoperative C2-7 angle. (**D**) C2-7 range of motion (ROM) was calculated as subtraction of C2-7 angle from extension position to flexion position (**D**), and change of C2-7 range of motion was calculated as (preoperative C2-7 ROM)—(postoperative C2-7 ROM)). (**D**) Spinal cord signal intensity change in magnetic resonance imaging (MRI) T2 weighted image were assessed because the intramedullary signal change had been reported as one of the imaging factors for prediction of surgical outcome (**E**, arrow).

*Imaging factors* Imaging factors including types of OPLL (continuous, segmental, mixed and localized types Fig. 1A)^27^, canal narrowing rate (thickness of OPLL at its peak level / antero-posterior diameter of corresponding spinal level (%) in lateral radiogram of cervical spine (Fig. 1B), post-operative change of C2-7 angle (C2-7 angle was measured as angle between inferior endplates of C2 and C7 vertebral bodies (Fig. 1C), and post-operative change of C2-7 angle was calculated as subtraction of postoperative C2-7 angle from preoperative C2-7 angle), change of C2-7 range of motion (ROM was calculated as subtraction of C2-7 angle from extension position to flexion position (Fig. 1D), and change of C2-7 range of motion was calculated as (preoperative C2-7 ROM)—(postoperative C2-7 ROM)). Spinal cord signal intensity change in magnetic resonance imaging (MRI) T2 weighted image were assessed because the intramedullary signal change had been reported as one of the imaging factors for prediction of surgical outcome (Fig. 1E)^28^.

**Figure 2:**
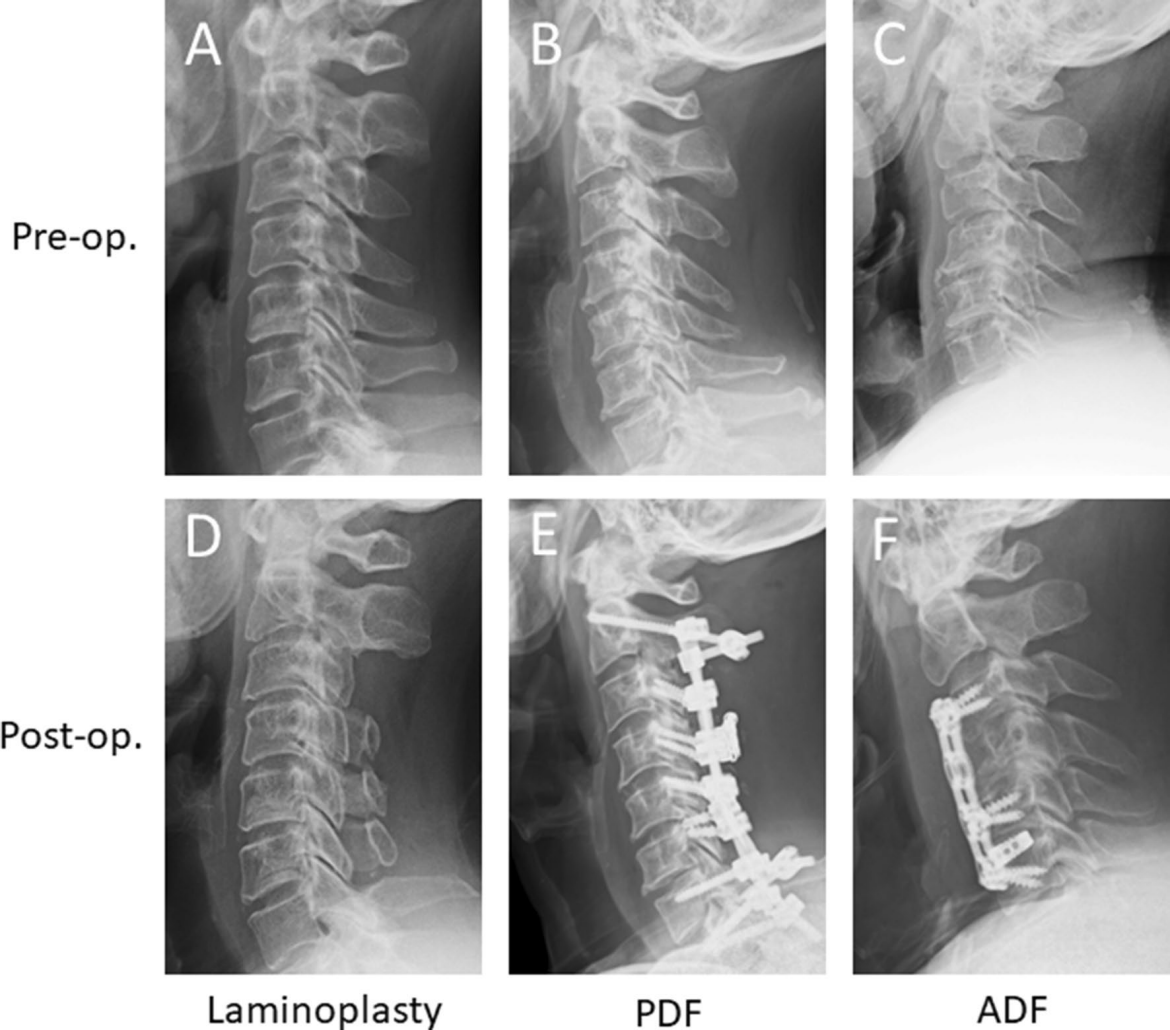
Representative images of each surgical procedures. Laminoplasty included open-door method and double-door method. There is no significant difference in any postoperative assessment between both procedures. Preoperative (**A**) and postoperative (**D**) radiograms of the OPLL patient received laminoplasty. Posterior recompression with instrumented fusion (PDF) was performed as combinatory procedure of laminoplasty or laminectomy followed by instrumented posterior fusion. Preoperative (**B**) and postoperative (**E**) radiograms of the OPLL patient received PDF. Anterior decompression with fusion (ADF) was performed as subtotal corpectomy with extirpation or floating of ossification foci followed by autologous bone graft with anterior plating. Preoperative (**C**) and postoperative (**F**) radiograms of the OPLL patient received ADF.

*Surgical factors*: Surgical factors including surgical procedures (laminoplasty, Fig. 2A), posterior decompression with instrumented fusion (PDF, Fig. 2B) and anterior decompression and fusion (ADF, Fig. 2C) and surgical approach (anterior and posterior) were evaluated.

Missing data were supplemented by the last observation carried forward method because the VAS score of neck pain was 46.2 ± 27.2 mm in 1 year after surgery and 48.2 ± 28.8 mm in 2 years after surgery, showing no significant difference between 2 time points.

First, we analysed the association of surgical procedures with post-operative neck pain. Pre- and post-operative VAS neck pain scores and the proportion of post-operative pain reduction were compared between surgical procedures including laminoplasty, PDF and ADF surgeries with Steel–Dwass analyses. Next, we performed uni-variate analyses followed by multi-variate analysis using stepwise logistic regression to elucidate the independent factors having a significant positive association with post-operative neck pain attenuation. Achievement of 50% or more post-operative neck pain reduction ratio was set as a response variable. The background factors for the patients as mentioned above, surgical factors, neurological factors, and imaging factors were set as explanatory variables. All the factors were checked the multicollinearity each other before univariate analyses.

Factors showing a *p*-value < 0.1 with initial uni-variate analyses were then analysed by stepwise logistic regression. Factors showing a *p*-value < 0.05 were determined as independent factors having a significant positive association with post-operative neck pain attenuation. Odds ration and 95% confidence interval was calculated for screened factors. Screened factors were then analysed using receiver-operator characteristic (ROC) curves to determine their cut-off values. All the statistical analyses were conducted with statistical analytics software JMP (version 12.0; SAS Institute, Cary, NC, USA) under the supervision by the biostatistician in our department (one of the co-author KF). Those statistical analyses were performed on data obtained 1 and 2 years after surgery.
